# A Study on the Mechanical Properties of Engineered Geopolymer Composites Based on Red Mud, Fly Ash, and Slag

**DOI:** 10.3390/polym18182271

**Published:** 2026-09-17

**Authors:** Shuo Xu, Yu Ling, Xin Luo, Jingyuan Wang, Yicong Zhong, Gai Chen

**Affiliations:** 1 Guangzhou Power Supply Bureau, Guangdong Power Grid Co., Ltd., Guangzhou 510620, China15915714058@163.com (Y.L.);; 2 School of Civil and Transportation Engineering, Guangdong University of Technology, Guangzhou 510006, China; 15766019812@163.com; 3 School of Future Transportation, Guangzhou Maritime University, Guangzhou 510725, China; zhongyicong2024@163.com; 4 School of Civil Engineering and Transportation, Foshan University, Foshan 528225, China

**Keywords:** engineered geopolymer composites, red mud, compressive properties, tensile properties

## Abstract

To promote the valorization of red mud (RM), a high-volume industrial solid waste, engineered geopolymer composites (EGC) were developed using a ternary binder comprising ground granulated blast-furnace slag (GGBS), fly ash (FA), and RM. Ten mixtures were designed using a simplex-centroid method, and the effects of binder composition on fresh-state flowability, axial compressive behavior, and axial tensile performance were systematically investigated. The results showed that GGBS was the dominant factor governing compressive strength. The mixture with the highest GGBS content, G50F50R0, achieved a maximum compressive strength of 111.7 MPa. Although RM incorporation reduced compressive strength, the strength remained approximately 70 MPa at an RM content of 30%, while the post-peak brittleness was noticeably mitigated. In tension, an appropriate RM content of 5–15% effectively reduced matrix toughness, enabling pronounced strain-hardening and multiple-cracking behavior through fiber bridging. Consequently, tensile ductility and strain energy density were substantially enhanced, with the ultimate tensile strain reaching up to 6.9%; however, excessive RM addition led to performance degradation. By overlaying multi-objective performance boundaries, an optimized composition range of 40–45% GGBS, 50–60% FA, and 0–8% RM was identified, enabling the simultaneous achievement of high strength, high toughness, and high energy absorption. These findings demonstrate the technical feasibility of using RM to produce EGC and provide a theoretical basis for the high-value utilization of RM.

## 1. Introduction

The cement industry is one of the major consumers of fossil fuels and sources of greenhouse gas emissions worldwide. It has been estimated that the production of ordinary Portland cement (OPC) accounts for approximately 5–10% of global anthropogenic carbon emissions [1,2,3]. In response to escalating climate-change challenges and increasing resource constraints, reducing carbon emissions from cement production and lowering the environmental footprint of the construction sector have become global priorities. Accordingly, the development of low-carbon binders as alternatives to conventional cement has emerged as a key pathway toward the sustainable transformation of the built environment [4,5,6,7]. Geopolymers, a class of alkali-activated materials, are produced by activating aluminosilicate-rich industrial by-products, such as fly ash (FA) and ground granulated blast-furnace slag (GGBS), with alkaline activators to form three-dimensional inorganic polymeric networks [8,9]. They can exhibit mechanical properties comparable to those of OPC-based materials, while requiring only approximately 40% of the energy and generating about 20% of the carbon emissions associated with OPC production [10,11]. Moreover, geopolymer technology enables the valorization of large-volume industrial solid wastes, offering substantial environmental benefits together with considerable potential for engineering applications.

Geopolymer materials have been increasingly developed into a range of construction materials, including geopolymer concrete [12,13], ultra-high-performance geopolymer concrete [9,14,15], and engineered geopolymer composites (EGC) [9,11,16,17,18]. Among these, EGC employ a geopolymer mortar matrix reinforced with short discrete fibers to regulate crack initiation and propagation, thereby mitigating the susceptibility of brittle cementitious materials to cracking under tensile stresses and shrinkage deformation [19]. With appropriate matrix–fiber interfacial design, EGC can exhibit pronounced tensile strain-hardening and multiple fine-cracking behavior, with tensile strain capacities typically exceeding 3% and crack widths controlled to approximately 100 μm [20,21], demonstrating excellent ductility and crack-control capability. Lao et al. [22] developed an EGC combining ultra-high strength with exceptional toughness, achieving a compressive strength of 140 MPa and a tensile strain capacity of 8%, thereby advancing the synergistic enhancement of strength and ductility in EGC. Studies on other high-performance cementitious composite systems have likewise shown that crack evolution is closely associated with the ductile response of the material or structural system [23].

Red mud (RM) is a large-volume industrial solid waste generated during alumina refining and is characterized by its reddish-brown color due to its high hematite content. China, the world’s largest alumina producer, generates more than 70 million t of alumina annually, with approximately 1.0–1.5 t of RM produced per tonne of alumina [24]. Consequently, the cumulative stockpile of RM in China has exceeded 600 million t. At present, RM is still predominantly disposed of through dammed storage, which not only occupies extensive land resources but also poses serious environmental risks because the continuous leaching of free alkalis may contaminate surrounding soils and groundwater [25,26]. Although exploratory applications of RM have been reported in metallurgical recovery, environmental remediation materials, and construction products, its high alkalinity, fine particle size, and low reactivity continue to hinder large-scale utilization. As a result, the current comprehensive utilization rate of RM in China remains as low as approximately 8% [27].

In recent years, geopolymer technology has opened new avenues for the high-value utilization of RM. Previous studies have shown that, under suitable alkaline activation conditions, RM can exhibit latent pozzolanic activity, enabling it to partially replace conventional reactive precursors and form cementitious materials with appreciable structural integrity [26]. Nevertheless, owing to its relatively low reactivity in alkaline environments, alkali-activated binders based solely on RM generally exhibit insufficient mechanical performance, which limits their engineering application [28]. Liu et al. [29] synergistically utilized RM and municipal solid waste incineration fly ash (MSWI FA) as a composite alkali-activating system to produce a high-performance one-part geopolymer. Their results showed that RM significantly enhanced the early-age reaction kinetics of the MSWI FA-based geopolymer and accelerated the formation and precipitation of reaction products. Consequently, the 28-day compressive strength reached 57.6 MPa, representing a 138% increase relative to the control mixture. To further improve engineering performance, RM is commonly combined with highly reactive industrial by-products, such as GGBS and FA, to achieve synergistic activation. Xu et al. [30] developed a GGBS–FA–RM-based EGC containing a high proportion of RM and exhibiting both high strength and high ductility, with a compressive strength exceeding 100 MPa and a tensile strain capacity above 5%. Their study further indicated that RM incorporation enhanced precursor reactivity, refined the microstructure, and increased compressive strength, although it adversely affected tensile performance to some extent.

Research on EGC incorporating RM in multicomponent precursor systems remains limited, particularly with respect to the synergistic effects of precursor proportions on mechanical performance and ductility. Moreover, the compositional complexity of such multicomponent binders makes conventional one-factor-at-a-time approaches inadequate for achieving simultaneous optimization of multiple performance objectives. Accordingly, in this study, FA, GGBS, and RM were used as ternary precursors, and ten EGC mixtures were designed using a simplex-centroid method. The effects of binder composition on fresh-state flowability, uniaxial compressive behavior, and axial tensile performance were systematically investigated. By superimposing multiple performance boundaries, an optimal composition range was identified to simultaneously achieve high strength, high ductility, and high energy-absorption capacity. The findings are expected to provide a theoretical basis and technical support for the large-scale application of RM in sustainable, high-toughness construction materials.

## 2. Materials and Methods

### 2.1. Raw Materials

**Figure 1:**
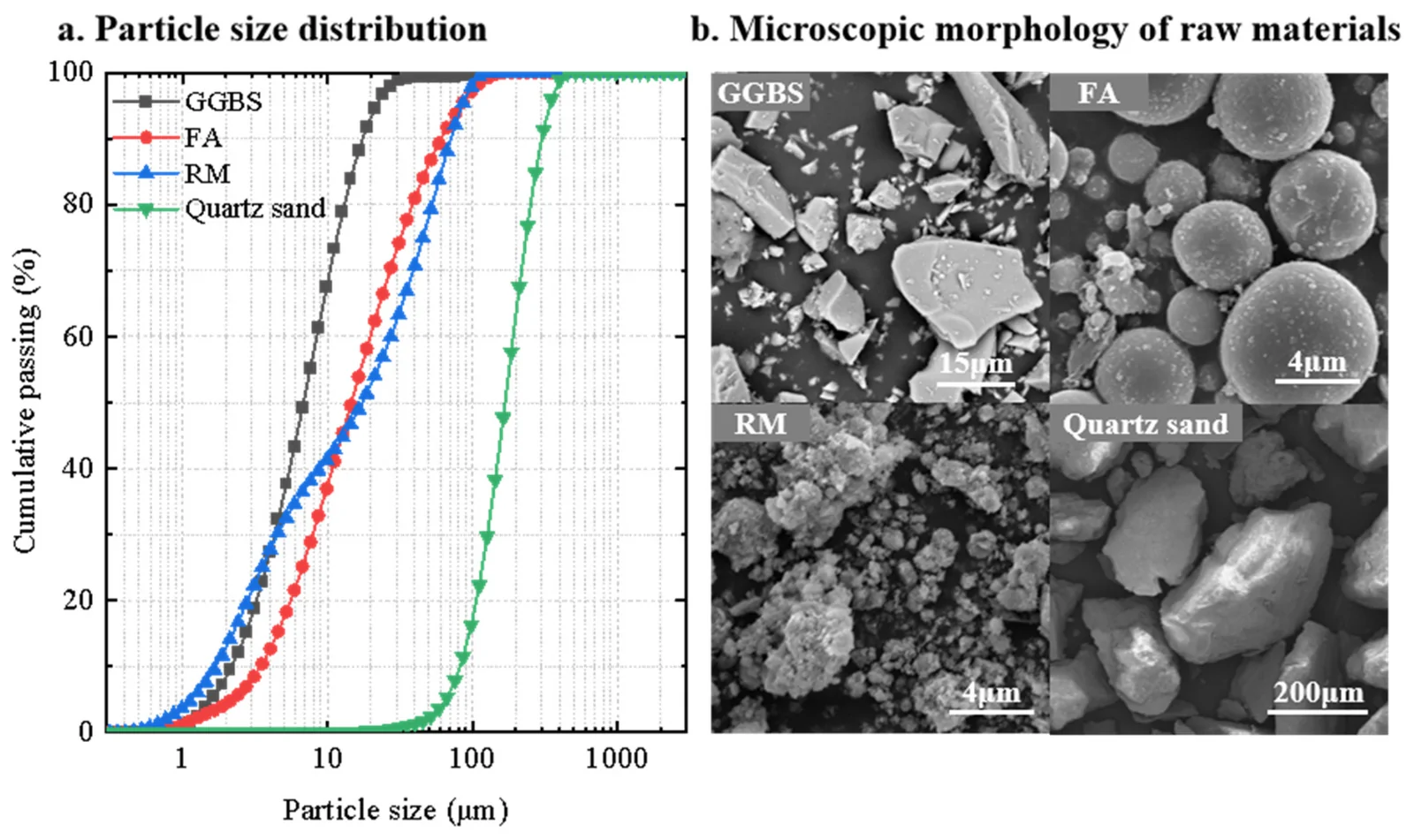
Particle-size distributions and micrographs of the binder materials and quartz sand.

The primary raw materials used in this study comprised binders, alkaline activators, aggregates, fibers, and water. The binder was a ternary blend of ground granulated blast-furnace slag (GGBS), fly ash (FA), and red mud (RM). Their microstructures and particle-size distributions are presented in Figure 1, and their chemical compositions are summarized in Table 1. RM was predominantly composed of irregular, fragmented particles with rough surfaces, often covered by nanoscale colloidal particles. Numerous densely distributed micropores were also observed, resulting in a relatively high specific surface area.

The alkaline activator was prepared by mixing a 10 mol/L sodium hydroxide solution with a water glass solution in a mass ratio of 1:2. It is prepared 24 h before use and then placed in a sealed container to stand and naturally cool to room temperature before being used. The inherent modulus of the water glass solution (the ratio of SiO_2_ to Na_2_O in moles) is 2.25, and the mass fractions of Na_2_O and SiO_2_ are 13.75% and 29.99% respectively. After mixing, the modulus of the alkaline activator was 1.2. Quartz sand with a particle-size range of approximately 101–310 μm was used as the aggregate, and its particle-size distribution is also shown in Figure 1. Ultra-high-molecular-weight polyethylene (UHMWPE, hereafter PE) fibers with a length of 12 mm and a diameter of 24 μm were used as reinforcement. PE fibers were provided by Suotite Special Line Co., Ltd. in Dongguan, China. The fibers had a tensile strength of 3000 MPa, an elastic modulus of 120 GPa, and a density of 0.97 g/cm^3^. In addition, barium chloride (BaCl_2_) was incorporated as a retarder to regulate the setting time. It reacts with SiO_3_^2−^ in the activator to form BaSiO_3_, which forms a dense coating on the surface of the precursor particles, preventing the contact between the alkali activator and the raw material particles, thereby prolonging the setting time [31].

### 2.2. Mix Proportions

**Figure 2:**
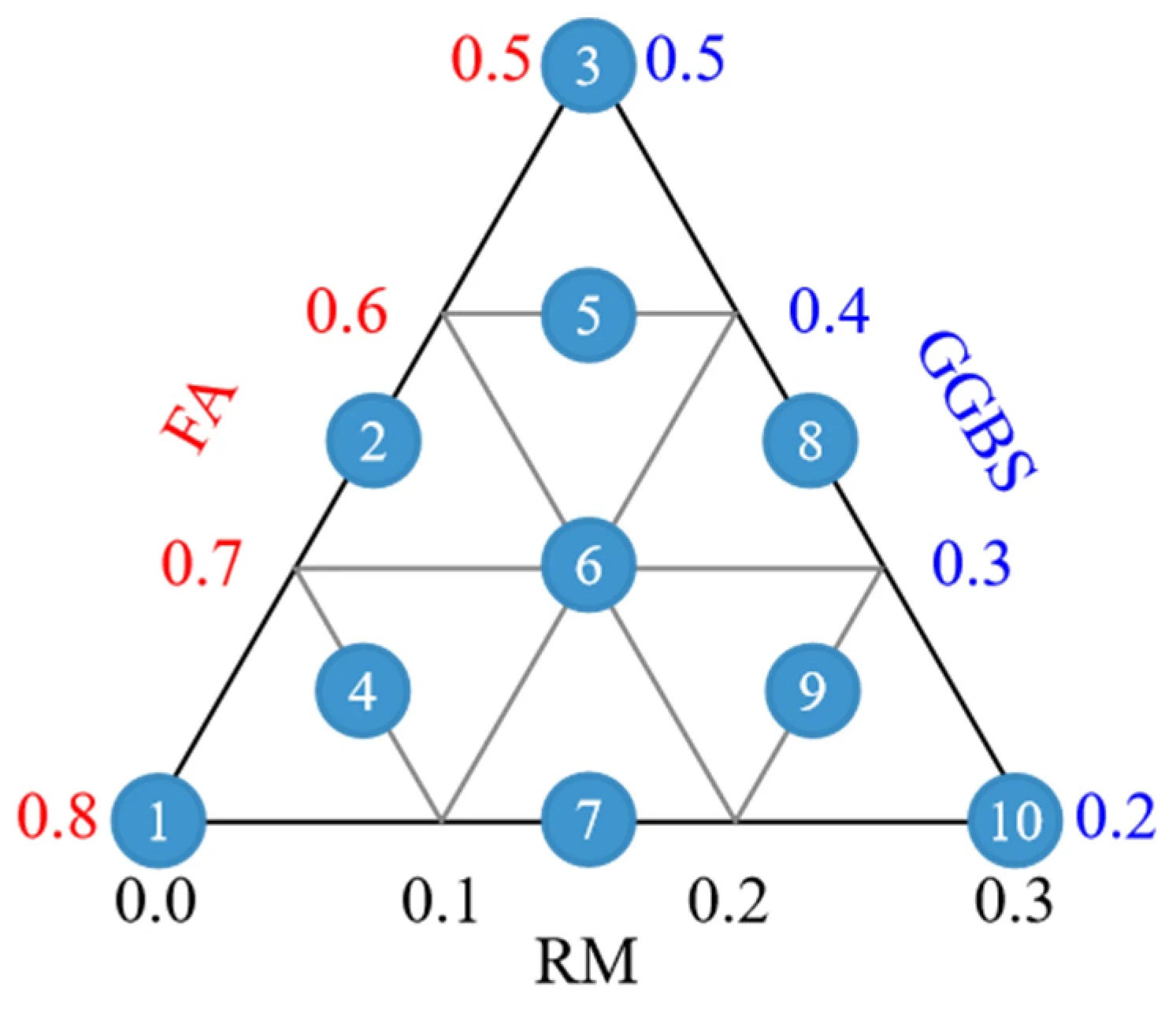
Binder compositions designed using the simplex-centroid method augmented with axial points.

A simplex-centroid design augmented with axial points was employed to formulate the fly ash–GGBS–RM ternary binder system and investigate the mechanical performance of the resulting engineered geopolymer composites under static loading. Based on the appropriate binder composition ranges reported in previous studies [30,32] and the screening of ternary precursor dosages through preliminary experiments, the mass fractions of GGBS, FA, and RM in the total binder were specified as 20–50%, 50–80%, and 0–30%, respectively. Three additional axial-point mixtures were incorporated to enhance the experimental design, yielding a total of ten binder compositions, as illustrated in Figure 2. The proportions of the remaining EGC constituents were kept constant: the water-to-binder ratio was 0.32, the alkali content was 6.8%, the alkali activator modulus was 1.2, the sand-to-binder ratio was 0.2, the PE fiber content was 2 vol.%, and the BaCl_2_ content was 1%. The detailed mixture proportions are provided in Table 2. The mix ratio includes two controlled alternative paths: (1) Keep the FA content constant at 50%, increase the RM while reducing GGBS by the same amount, to examine the effect of RM replacing GGBS; (2) Keep the GGBS content constant at 20%, increase the RM while reducing FA by the same amount, to examine the effect of RM replacing FA.

**Figure 3:**
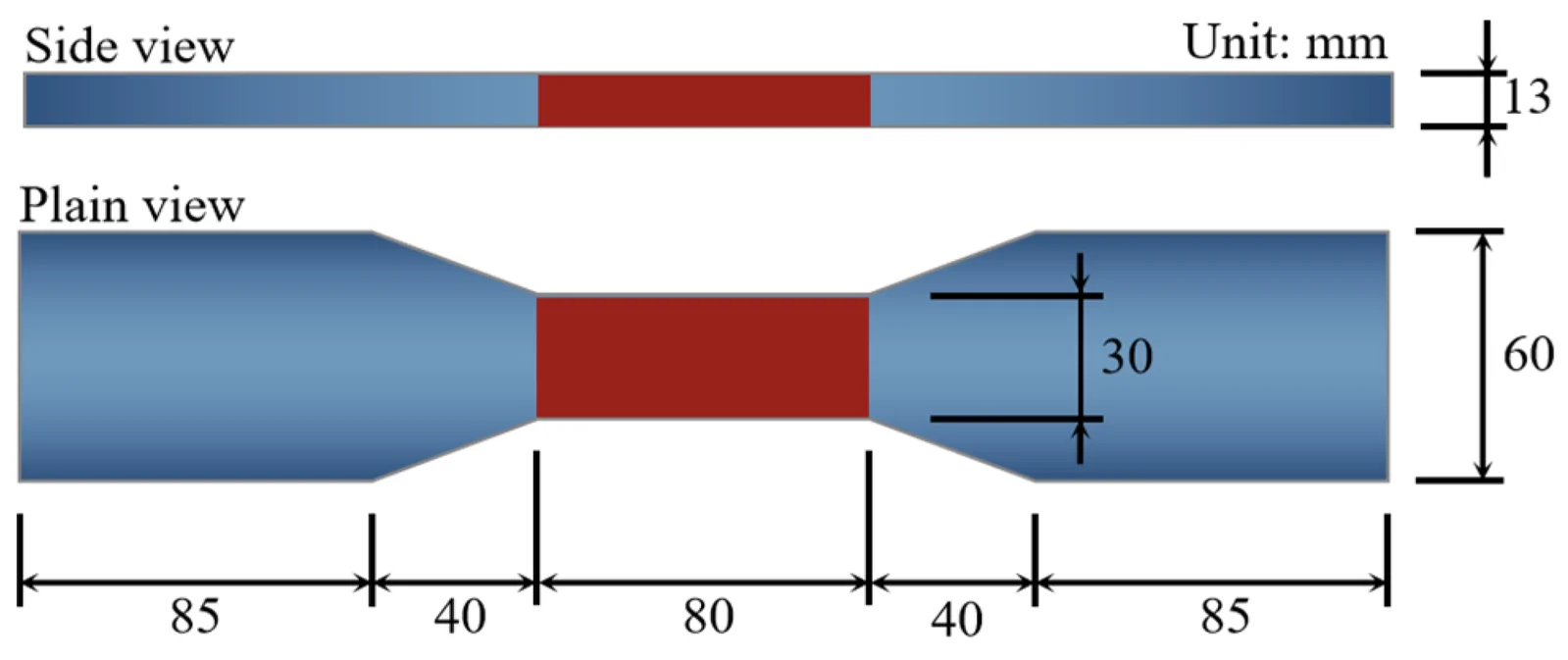
Specimen for axial tensile testing.

The specimens comprised cylindrical specimens measuring φ50 mm × 100 mm for uniaxial compression tests and dog-bone-shaped specimens for axial tensile tests, as shown in Figure 3. The EGC mixtures were prepared as follows. First, the dry constituents, including RM, FA, GGBS, quartz sand, and BaCl_2_, were introduced into a planetary mixer and blended at low speed for 3 min. The premixed alkaline solution and water were then added at a constant rate while low-speed mixing was maintained for an additional 3 min to obtain a homogeneous matrix. Subsequently, the pre-dispersed fibers were gradually incorporated, followed by a further 3 min of mixing. The fresh mixture was then cast into molds coated with a release agent and compacted on a vibrating table. Immediately after casting, all specimens were sealed with plastic film. After 24 h, they were demolded and transferred to a standard curing room maintained at 20 ± 2 °C and a relative humidity of at least 90%, where they were cured for 28 d prior to testing.

### 2.3. Experimental Methods

The workability of the fresh EGC mixtures was evaluated in accordance with GB/T 2419-2005 [33] using a truncated-cone flow test. After removal of the mold, the maximum spread diameters of the fresh mixture were measured along two mutually perpendicular directions using calipers, and their average value was taken as the flowability index.

**Figure 4:**
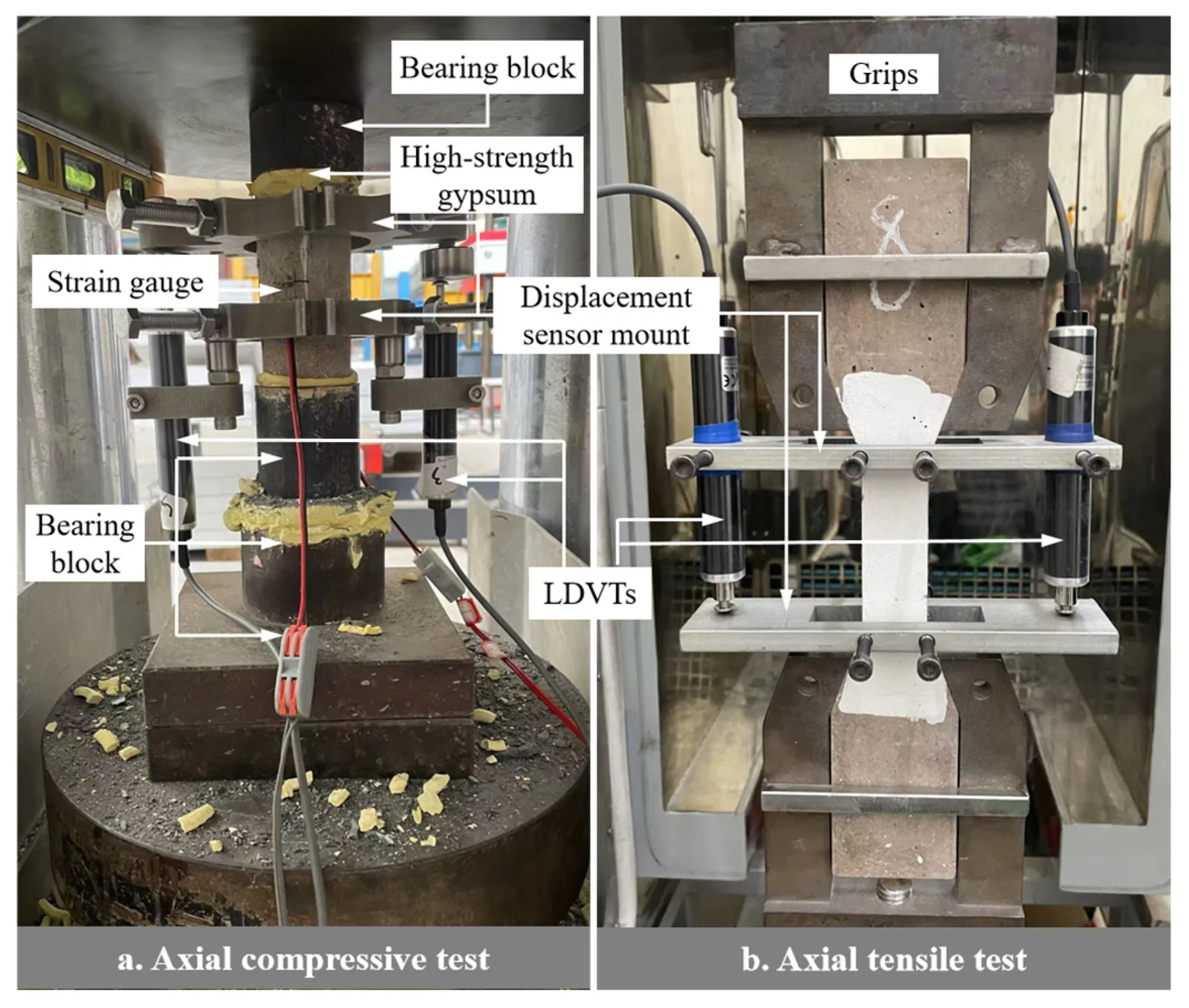
Experimental setups for the uniaxial compression tests and axial tensile tests.

The uniaxial compression tests were conducted in accordance with ASTM C39/C39M-24 [34]. Before testing, the top and bottom surfaces of each specimen were ground, cleaned to remove loose particles, and capped with gypsum to ensure uniform load transfer and minimize eccentric loading. Axial deformation was measured using symmetrically arranged linear variable differential transformers (LVDTs) secured to collars at both ends of the specimen, together with axial strain gauges mounted on two opposite sides of the specimen. The LVDTs had a measurement range of 50 mm. Loading was applied using a MATEST electrohydraulic servo testing machine under displacement control at a constant rate of 0.18 mm/min. Test data were recorded using a TDS-540 static data acquisition system at a sampling frequency of 1 Hz. The compression test setup is shown in Figure 4a.

The axial tensile tests were performed in accordance with the JSCE recommendations [35]. Universal joints were installed at both the upper and lower grips to minimize eccentric tensile loading. Before testing, one surface of each dog-bone-shaped specimen was coated with white paint to facilitate subsequent observation of crack distribution. Specimen deformation was measured using LVDTs mounted on both sides of the specimen over an 80 mm gauge length in the central region. Data were recorded at a sampling frequency of 1 Hz. The tests were conducted under displacement control at a loading rate of 0.5 mm/min. The tensile test setup and specimen dimensions are presented in Figure 4b.

The micro-test was conducted using a Phenom Pharos G2 scanning electron microscope (Fonar Scientific Instruments (Shanghai) Co., Ltd., Shanghai, China) at an acceleration voltage of 15 kV to observe and characterize the microstructure of the sample. Before the observation, the specimen was cut into small pieces and dried. Subsequently, the surface of the sample was gold-coated in a vacuum to enhance its conductivity. The fiber failure mode of the loaded specimen sample was studied through the scanning electron microscope.

## 3. Results and Discussion

### 3.1. Flowability

**Figure 5:**
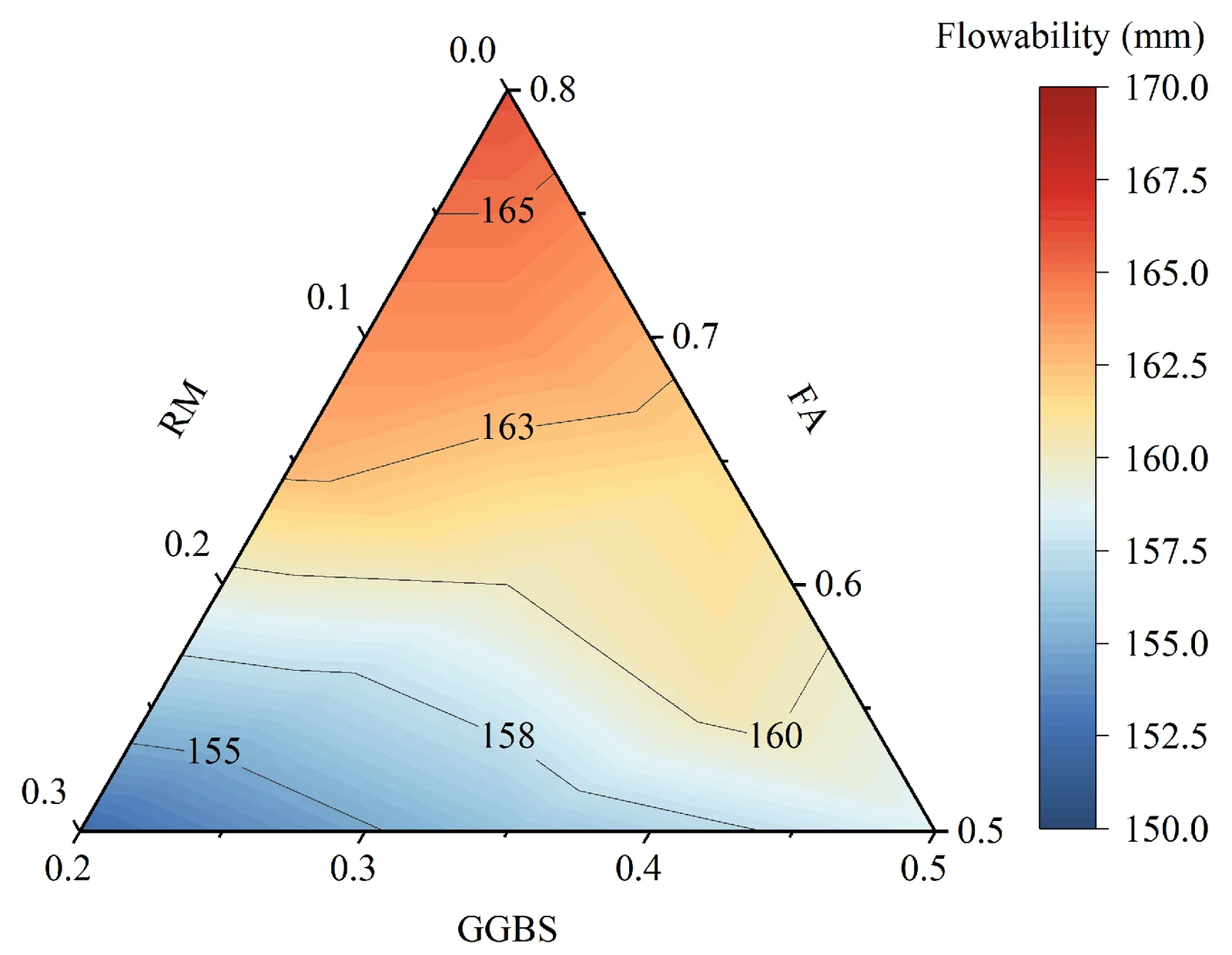
Flowability test results of the EGC mixtures.

The flowability of fresh mixtures is critical to construction efficiency and casting quality. Adequate flowability facilitates compaction and reduces the formation of entrapped air voids and other casting defects, thereby contributing to improved overall material performance. Figure 5 presents the flowability results of the EGC mixtures. Owing to the ball-bearing effect of the predominantly spherical FA particles, the flowability of EGC generally increased with increasing FA content [36]. By contrast, the influence of the irregularly shaped GGBS particles was less pronounced and depended largely on the corresponding variations in the other binder constituents.

Among all mixtures, G20F80R0, which contained no RM, exhibited the highest flow spread of 166 mm, whereas G20F50R30, with the highest RM content, showed the lowest value of only 152.5 mm. Overall, RM incorporation adversely affected EGC flowability, with the flow spread decreasing progressively as the RM proportion increased. The morphology of RM particles lies between the spherical shape of FA and the irregular angular shape of GGBS. In addition, numerous nanoscale particles adhere to the RM surfaces, resulting in greater surface roughness and a higher specific surface area. These physical characteristics, together with the disturbance of the original particle-packing structure caused by RM addition, may increase the water demand and interparticle friction of the fresh matrix, thereby reducing its flowability.

The relationships between the mixture variables and material properties in the simplex-centroid design can be expressed by Equation (1):

(1) $$Y\left(x_{1},x_{2},x_{3}\right)=\beta_{1}x_{1}+\beta_{2}x_{2}+\beta_{3}x_{3}+\beta_{12}x_{1}x_{2}+\beta_{13}x_{1}x_{3}+\beta_{23}x_{2}x_{3}+\beta_{123}x_{1}x_{2}x_{3}$$

where $x_{i}(i=1,2,3)$ denotes the mass fractions of GGBS, FA, and RM, respectively, while $\beta_{i}$, $\beta_{ij}$ and $\beta_{ijk}(i,j,k=1,2,3)$ are the coefficients representing the individual and interaction effects of the binder components. By fitting the experimental data, the relationship between binder composition and EGC flowability was obtained, as expressed in Equation (2), with a coefficient of determination of $R^{2}=0.981$:

(2) $$Flowability=157x_{1}+173x_{2}+8x_{3}-24x_{1}x_{2}+171x_{1}x_{3}+219x_{2}x_{3}-249x_{1}x_{2}x_{3}$$

As indicated by Equation (2), GGBS and FA exert positive effects on flowability, and their contributions are substantially greater than that of RM. The ternary interaction coefficient $\beta_{123}=-249$ suggests a pronounced adverse interaction among the three binder components. This reduction in flowability may be attributed to changes in the particle-size distribution. As shown in Figure 1, the particle-size range of RM overlaps those of both GGBS and FA, containing particles finer than GGBS as well as particles coarser than FA. Consequently, the incorporation of RM disrupts the original particle-size distribution characterized by a broad overall range but relatively narrow local distributions, thereby increasing the resistance to flow and reducing the flowability of the fresh matrix.

### 3.2. Compressive Behaviors

#### 3.2.1. Compressive Stress–Strain Curves

**Figure 6:**
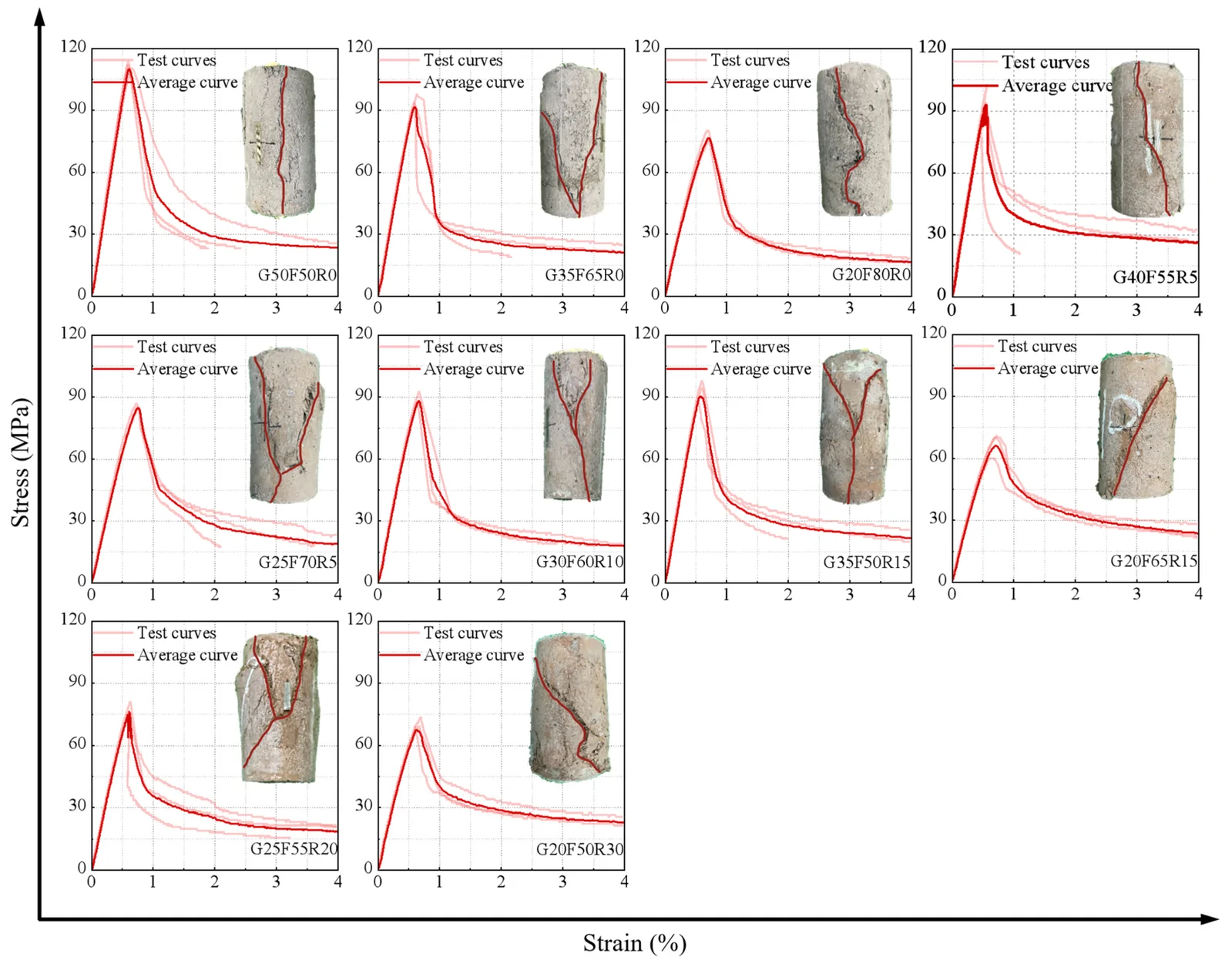
Compressive stress–strain curves and corresponding failure modes of the EGC mixtures.

Figure 6 presents the compressive stress–strain curves and corresponding failure modes of the EGC mixtures. As shown, the complete compressive stress–strain response was approximately linear prior to the peak, with no pronounced degradation in elastic stiffness. After reaching the peak stress, a dominant crack developed; however, due to fiber bridging, the stress decreased gradually with increasing strain, indicating favorable post-peak deformability.

A comparison of the curves for G50F50R0, G35F50R15, and G20F50R30 further shows that, when the FA content was kept constant, increasing the RM content while decreasing the GGBS content led to a progressive reduction in compressive strength, whereas the abrupt post-peak stress drop became less pronounced. This indicates that when the FA content remains at 50%, substituting an equal amount of RM for GGBS can to some extent alleviate the brittleness of the material, while increasing the proportion of GGBS is the key to enhancing the axial compressive strength of the material. A comparison among G20F80R0, G20F65R15, and G20F50R30 indicates that, at a constant GGBS content, the compressive strength decreased as the RM proportion increased. This behavior is mainly attributed to the rough surface texture of RM particles and their lower contents of reactive silica and alumina [37].

Inspection of the failure patterns in Figure 6, together with the observed test phenomena and previous findings [9], indicates that the specimens exhibited two principal failure modes:

(1) Longitudinal splitting failure. Fine microcracks first developed in the central region of the specimen and progressively coalesced into a dominant through-crack as the load increased. The crack propagated predominantly along the loading direction, producing a distinct vertical splitting pattern without significant surface spalling. This failure mode was generally observed in specimens with relatively high compressive strength, such as G50F50R0 and G40F55R5. Their superior strength can be attributed to the more extensive reaction of the binder constituents, which promoted the formation of a dense calcium aluminosilicate hydrate (C–A–S–H) gel network and a compact matrix structure [38]. During loading, the matrix therefore dominated the load-bearing response, while the contribution of fiber bridging remained comparatively limited.
(2) Inclined shear failure. In this mode, the dominant crack formed at an oblique angle to the specimen axis, exhibiting the characteristic features of shear failure. This behavior was primarily observed in specimens with relatively low compressive strength, including G20F80R0, G20F50R30, and G20F65R15. These mixtures contained only 20% GGBS; consequently, the limited availability of calcium ions restricted the formation of C–A–S–H gel and resulted in a weaker matrix [38]. Under these conditions, the crack-bridging effect of the fibers became more evident.

**Figure 7:**
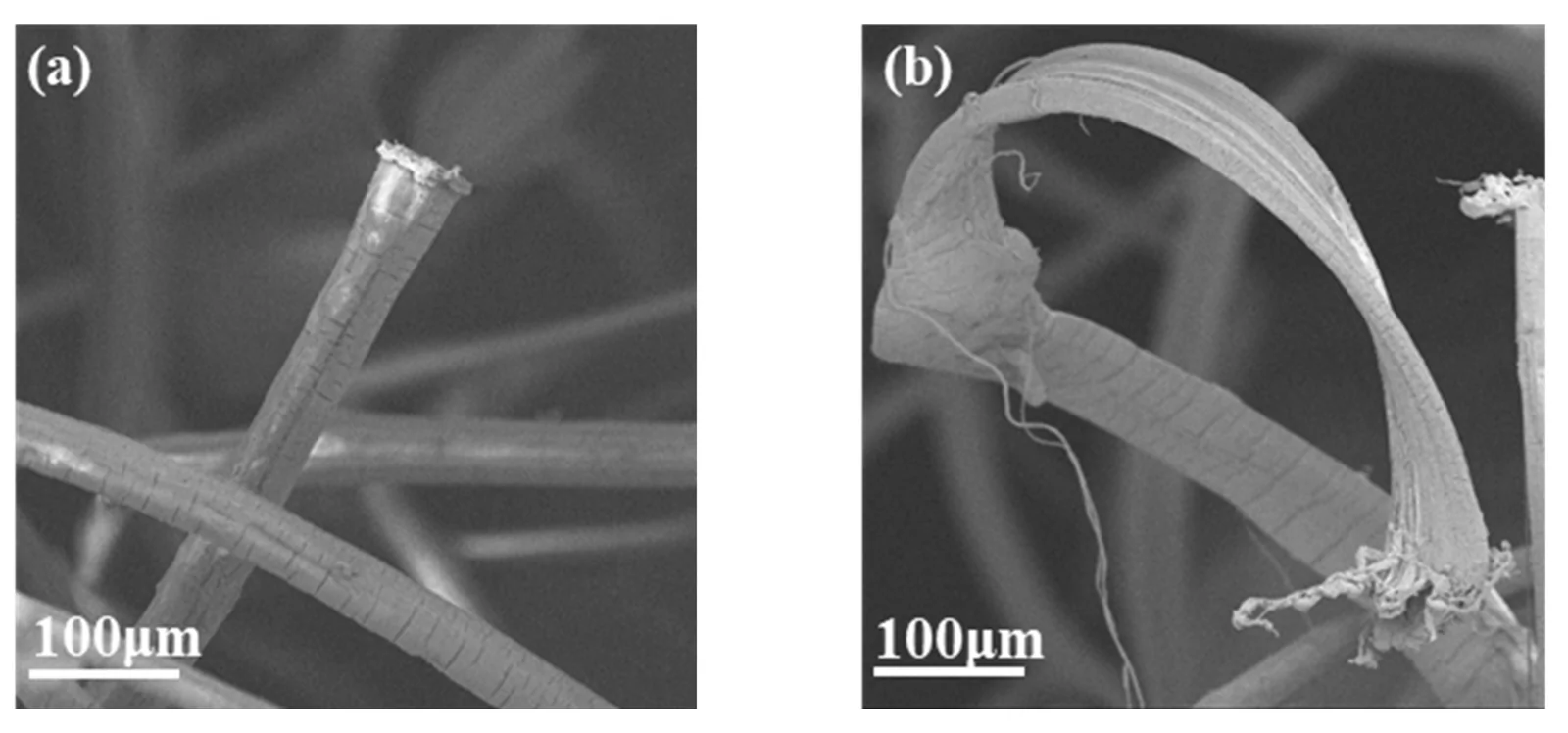
Failure modes of fibers: (**a**) Fiber pull-out failure. (**b**) Fiber breakage failure.

As shown in Figure 7, SEM observations were conducted on the fracture surfaces of the main cracks of the damaged specimens. The results indicated that the failure mode of the fibers varied with the matrix strength in two typical patterns: for the mix ratio with lower axial compression strength (such as G20F50R30), the fracture morphology of the fibers was smooth and was accompanied by a small amount of matrix debris. The cross-sectional morphology was basically the same as the original fibers (Figure 7a), indicating that during the expansion of the cracks, the fibers detached from the matrix and were pulled out from the matrix as a whole, which is a pull-out failure; for the mix ratio with higher compressive strength (such as G50F50R0), the fracture of the fibers presented a clear tearing feature and was accompanied by matrix debris (Figure 7b), indicating that during the bridging process, the tensile stress on the fibers reached their tensile strength and they were pulled apart, which is a pull-off failure. The differences in the failure modes of the fibers explained the differences in the post-peak responses of different strength mix ratios: when the matrix strength decreased, the failure mode of the fibers changed from pull-off to pull-out, the energy dissipation process of fiber bridging was prolonged, and the brittleness of the material after the peak was alleviated.

#### 3.2.2. Compressive Performance

The compressive properties of the EGC mixtures, including compressive strength (*f_c_*), peak strain (*ε_c_*), and elastic modulus (*E*), were obtained from the uniaxial compression tests. The elastic modulus was calculated in accordance with ASTM C469/C469M-22 [39] using Equation (3):

(3) $$E=\frac{\sigma_{1}-\sigma_{2}}{\varepsilon_{1}-0.00005}$$

where σ_1_ is the stress corresponding to 40% of the ultimate load, σ_2_ is the stress corresponding to a strain of 0.00005, and ε_1_ is the strain corresponding to σ_1_.

The detailed compressive properties are summarized in Table 3. The compressive strengths of the EGC mixtures with different binder compositions ranged from approximately 60 to 120 MPa. G50F50R0 exhibited the highest compressive strength of 111.7 MPa, whereas G20F50R30 showed the lowest value of 70.3 MPa, representing a reduction of 37.1%. The comparative analysis along two controlled alternative paths indicates that the effect of RM on the compressive strength of EGC depends on the component being replaced. When the FA content is constant at 50%, the compressive strength significantly decreases when GGBS is replaced by RM. For every 10% substitution of GGBS by RM, the strength decreases by approximately 13.8 MPa on average; meanwhile, when the FA content is constant at 20%, the decline in compressive strength slows down when RM replaces FA. Furthermore, the mixture containing 30% RM still achieved a compressive strength of approximately 70 MPa, demonstrating sufficient strength for a broad range of structural applications. The peak strain generally increased with increasing FA and RM contents. As shown in Table 3, the elastic modulus of the EGC mixtures ranged from approximately 12 to 21 GPa. G50F50R0 exhibited the highest elastic modulus of 20.9 GPa, whereas G20F65R15 had the lowest value of 12.0 GPa, corresponding to a reduction of 42.6%. Overall, the elastic modulus and compressive strength exhibited similar variations with binder composition, both decreasing progressively as the GGBS proportion decreased.

**Figure 8:**
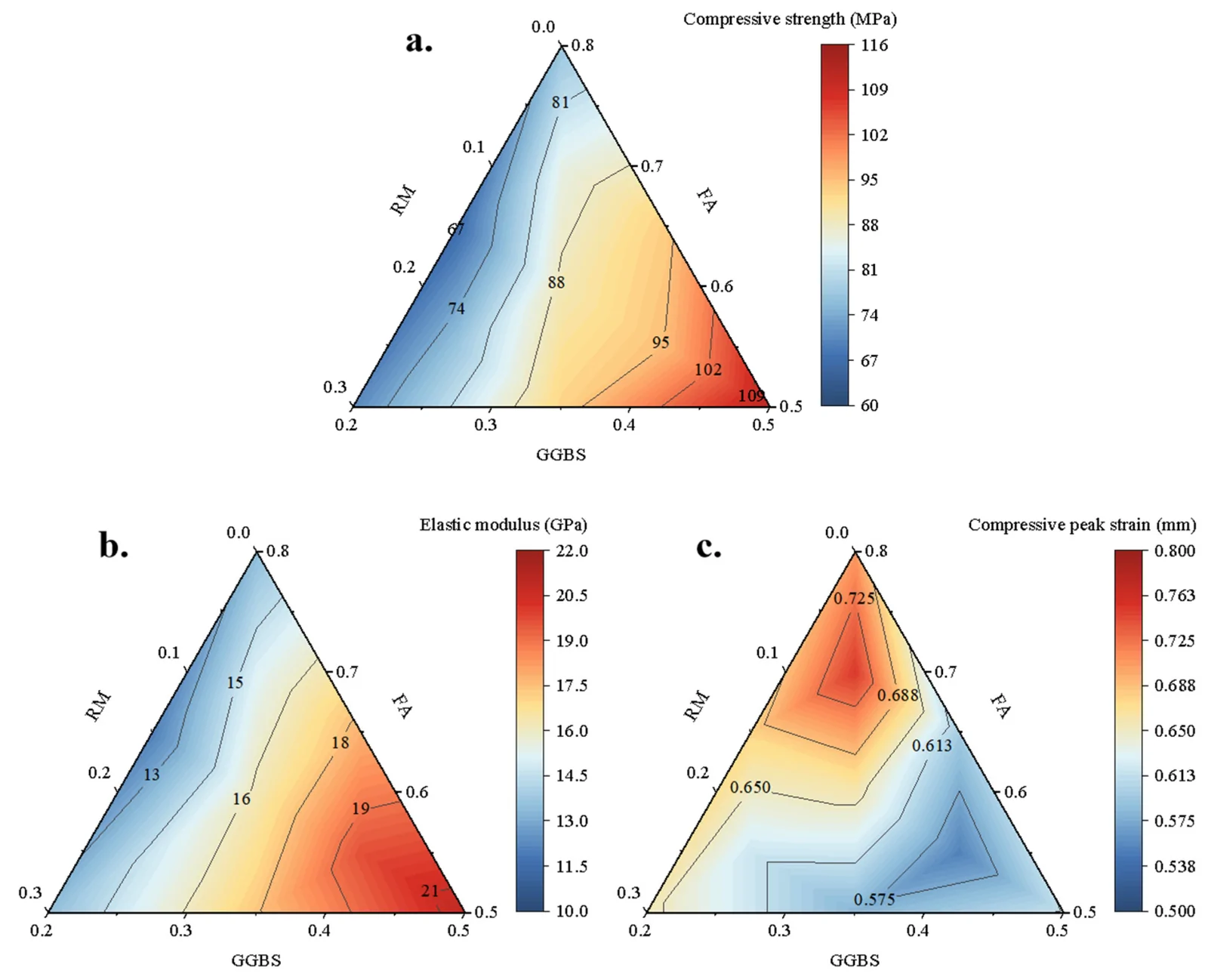
Contour plots of the axial compressive properties of the EGC mixtures: (**a**) compressive strength; (**b**) elastic modulus; and (**c**) peak compressive strain.

The contour plots of the compressive performance parameters derived from Table 3 are presented in Figure 8. As shown, the compressive strength of the EGC mixtures was strongly influenced by the proportions of GGBS and RM in the binder. Both compressive strength and elastic modulus exhibited an approximately linear positive correlation with the GGBS content and increased progressively as the RM and FA contents decreased. This behavior can be attributed to the higher reactivity of GGBS relative to RM and FA, owing to its richer content of reactive silica and alumina. Under alkaline activation, GGBS promotes the formation of dense calcium silicate hydrate (C–S–H) and calcium aluminosilicate hydrate (C–A–S–H) gels, thereby enhancing matrix compactness and, in turn, improving compressive strength.

Although increasing the GGBS proportion in the precursor system significantly enhances the compressive strength of the material, this does not imply that the GGBS content can be increased without limit. Excessively high GGBS contents may impair fresh and early-age performance, such as flowability and setting behavior [40]. By fitting the experimental data, the relationships between the compressive performance indices and the binder composition were established, as expressed in Equations (4)–(6):

(4) $$\sigma_{c}=175x_{1}+60x_{2}+602x_{3}-29x_{1}x_{2}-2167x_{1}x_{3}-1176x_{2}x_{3}+4456x_{1}x_{2}x_{3}$$

(5) $$E=21x_{1}+5x_{2}+123x_{3}+32x_{1}x_{2}-402x_{1}x_{3}-229x_{2}x_{3}+828x_{1}x_{2}x_{3}$$

(6) $$\varepsilon_{c}=1.5x_{1}+1.1x_{2}+5.8x_{3}-2.7x_{1}x_{2}-28x_{1}x_{3}-9.9x_{2}x_{3}+50.4x_{1}x_{2}x_{3}$$

### 3.3. Tensile Behaviors

#### 3.3.1. Tensile Failure Modes

**Figure 9:**
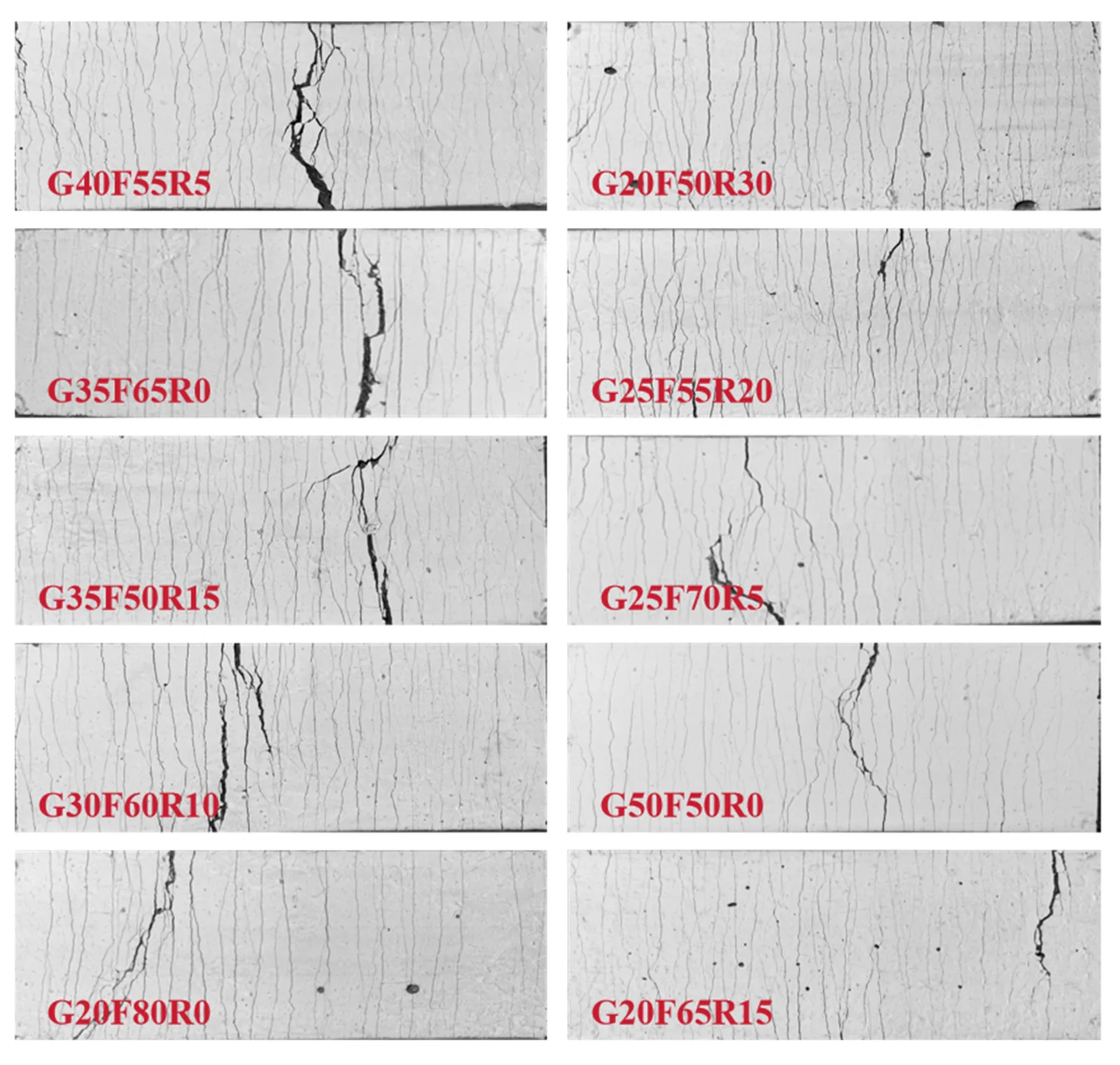
Tensile failure patterns of the EGC specimens.

Figure 9 presents the tensile failure patterns of the EGC specimens with different binder compositions, together with the crack distributions within the central gauge region. All specimens exhibited multiple fine cracking; however, the crack widths and crack densities varied markedly with binder composition. To quantify these differences, an optical microscope was used to measure the crack width (*w_c_* (μm)), and the number of cracks (*N_c_*) intersecting the longitudinal centerline within the 80 mm gauge length of each failed specimen. The average width of all measured cracks within the gauge region was taken as the representative crack width of the specimen, while the average crack spacing (*x_c_* (mm)), was calculated as *x_c_* = 80/*N_c_*. The resulting crack parameters are summarized in Table 4.

As shown in Table 4, the EGC specimens with different binder compositions exhibited pronounced differences in crack characteristics, primarily owing to the combined effects of matrix properties and fiber reinforcement. Nevertheless, the average crack spacing of all specimens remained below 2.3 mm, while the average crack width was controlled within 100 μm. These results confirm that the developed composites possess crack-control capability comparable to that of conventional engineered cementitious composites (ECC).

**Figure 10:**
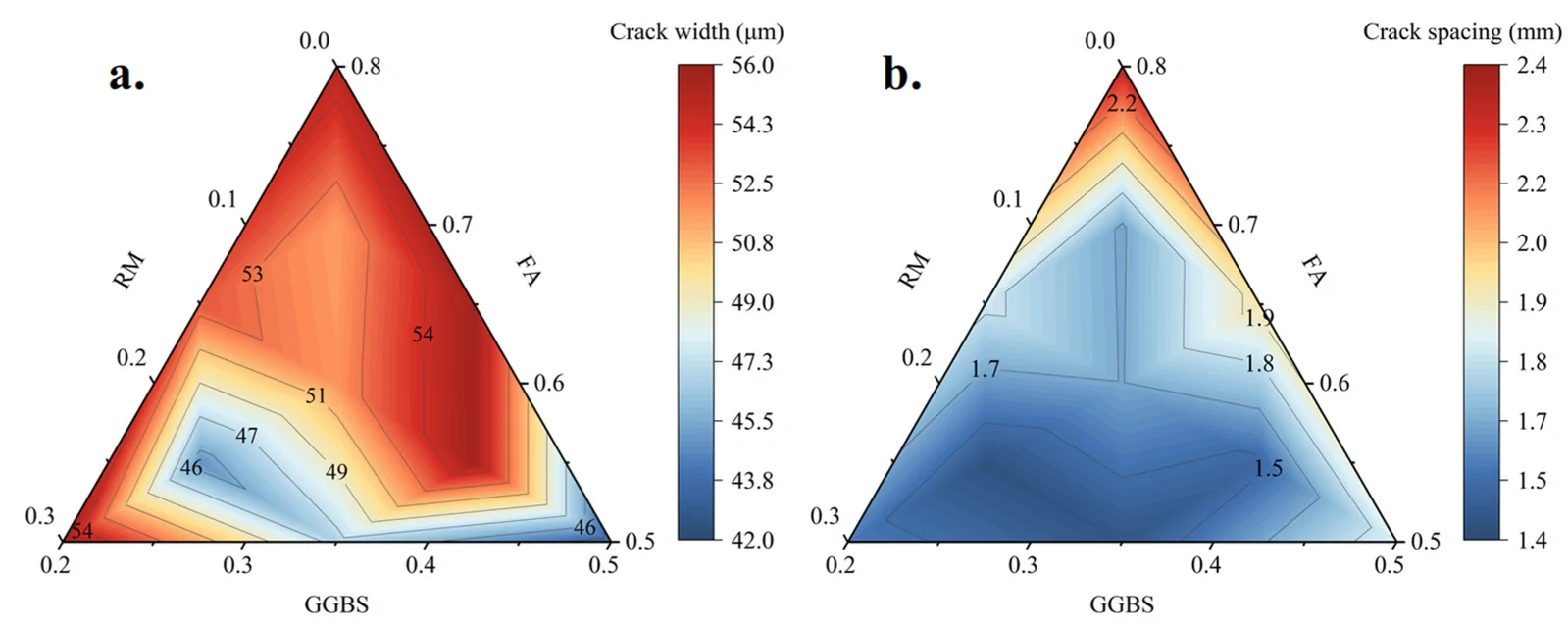
Tensile crack parameters of the EGC specimens: (**a**) crack width; (**b**) crack spacing.

To more clearly illustrate the differences in cracking behavior among the EGC mixtures, the data in Table 4 are plotted in Figure 10. As shown, mixtures containing 5–15% RM, such as G40F55R5 and G25F70R5, generally exhibited reduced crack widths. When the FA content was kept constant and the RM proportion was further increased to 30% (G20F50R30), the surface cracking characteristics changed markedly: the average crack width increased substantially, while the number of cracks tended to decrease. Compared with G35F50R15, G20F50R30 therefore exhibited distinctly poorer crack-control performance.

These results indicate that a moderate increase in the RM proportion can improve the crack-control capability of EGC within an appropriate range. Once the RM content exceeds a critical level, however, further addition becomes detrimental. A small amount of RM may introduce additional microstructural heterogeneities into the matrix, reducing its resistance to crack initiation and promoting the formation of a greater number of distributed cracks during tensile loading. By contrast, at high RM contents, excessive inert constituents, such as unreacted Fe_2_O_3_, occupy part of the binder volume and restrict the formation of effective reaction products [41]. The resulting reduction in matrix strength causes premature failure, leading to fewer but wider cracks.

#### 3.3.2. Tensile Stress–Strain Curves

The tensile stress–strain response of EGC can be broadly divided into four stages. The first is the elastic stage, during which the stress increases approximately linearly with strain until the first-cracking point is reached. In this stage, the tensile load is carried primarily by the matrix. The second stage involves stable crack propagation and strain hardening. Once a crack forms, fibers crossing the crack plane are mobilized, and the fiber-bridging stress exceeds the first-cracking strength of the matrix. Consequently, the crack remains stable while the tensile stress gradually increases with strain, producing a characteristic serrated response associated with multiple cracking and strain-hardening behavior. This is followed by a crack-saturation stage, during which the formation of new cracks slows, the stress–strain curve exhibits an upward trend, and the apparent stiffness increases. Finally, the specimen enters the strain-softening stage. At this point, the number of cracks becomes essentially stable, one dominant crack progressively widens, and the tensile stress decreases rapidly until failure.

**Figure 11:**
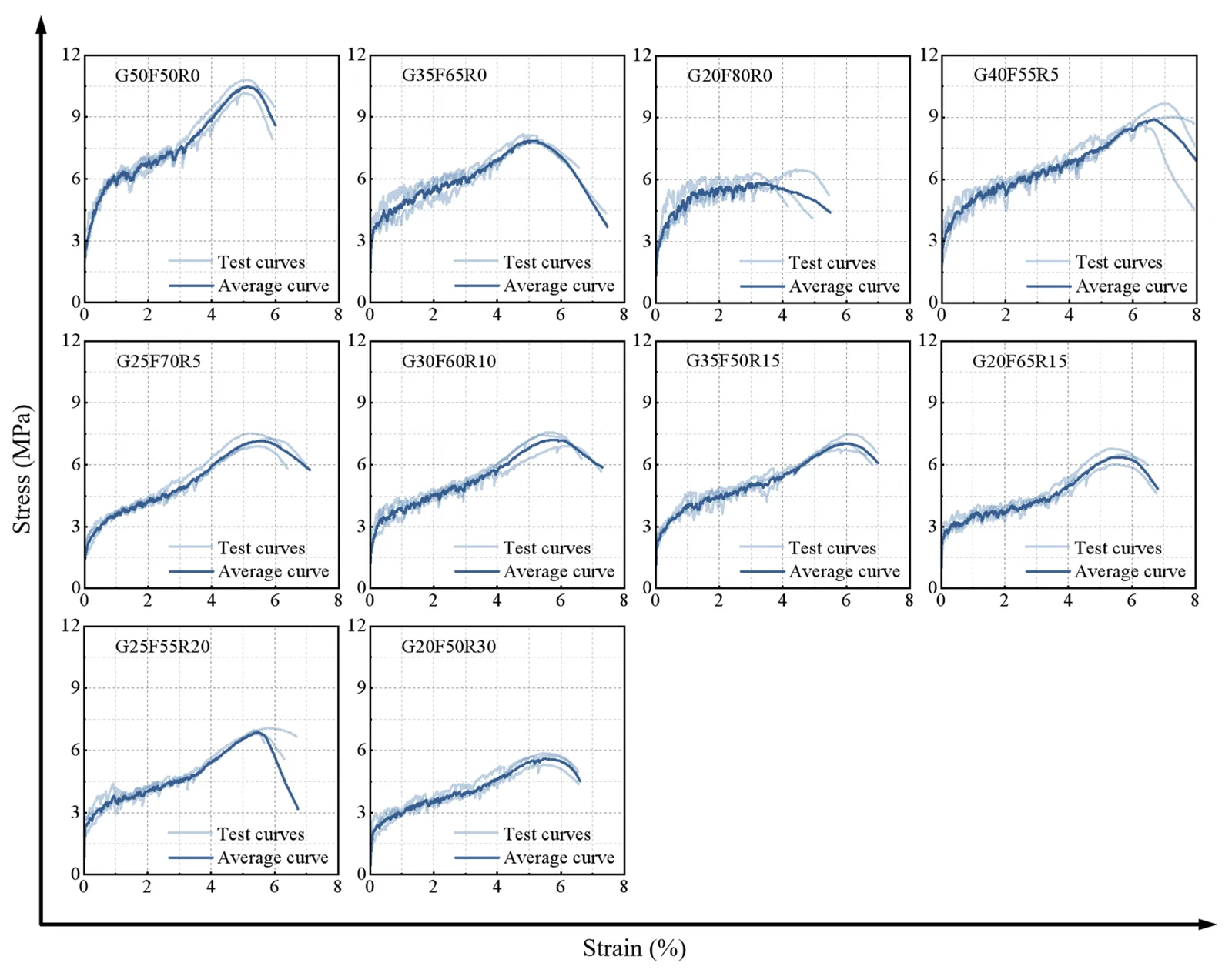
Axial tensile stress–strain curves of the EGC mixtures.

As shown in Figure 11, all specimens except G20F80R0 experienced the crack-saturation stage. This indicates that an excessively high FA-to-GGBS ratio is unfavorable for the full development of fiber-bridging capacity, causing premature tensile failure before the fibers can be effectively mobilized. By comparison, at the same GGBS content, partially replacing FA with RM produced little change in the tensile strength of G20F65R15 and G20F50R30, but substantially enhanced their tensile deformation capacity. This result highlights the beneficial role of RM in reducing matrix toughness and facilitating crack initiation and multiple-crack development in EGC. Figure 11 further shows that tensile strength generally increased with increasing GGBS content, with G50F50R0 exhibiting the highest tensile strength.

#### 3.3.3. Tensile Performance

The tensile properties extracted from Figure 11 are summarized in Table 5, including the first-cracking strength (*σ_tc_*), tensile strength (*σ_tu_*), ultimate tensile strain (*ε_tu_*), and strain energy density (*g_se_*). The tensile strength of the EGC mixtures with different binder compositions ranged from approximately 6 to 11 MPa, indicating a pronounced influence of binder proportion on tensile resistance. Among all mixtures, G50F50R0 exhibited the highest tensile strength of 10.47 MPa, whereas G20F50R30 showed the lowest value of only 5.60 MPa, representing a reduction of 46.5% relative to G50F50R0.

The ultimate tensile strain of the EGC mixtures varied approximately from 3% to 7%, and all values exceeded 3%, satisfying the requirement for high-ductility cementitious composites. The highest ultimate tensile strain was obtained for G40F55R5, reaching 6.90%, while the lowest value was observed for G20F80R0 at only 3.54%, which was 48.7% lower than that of G40F55R5. In terms of first-cracking strength, the minimum value of 1.61 MPa was recorded for G35F50R15, further confirming the effect of RM in reducing matrix toughness [42].

As a key indicator of the energy-absorption capacity of the material, the tensile strain energy density is governed jointly by the first-cracking strength, tensile strength, and ultimate tensile strain. The strain energy density of the EGC mixtures with different binder compositions ranged from approximately 180 to 450 kJ/m^3^, exhibiting a trend similar to that of tensile deformation capacity.

**Figure 12:**
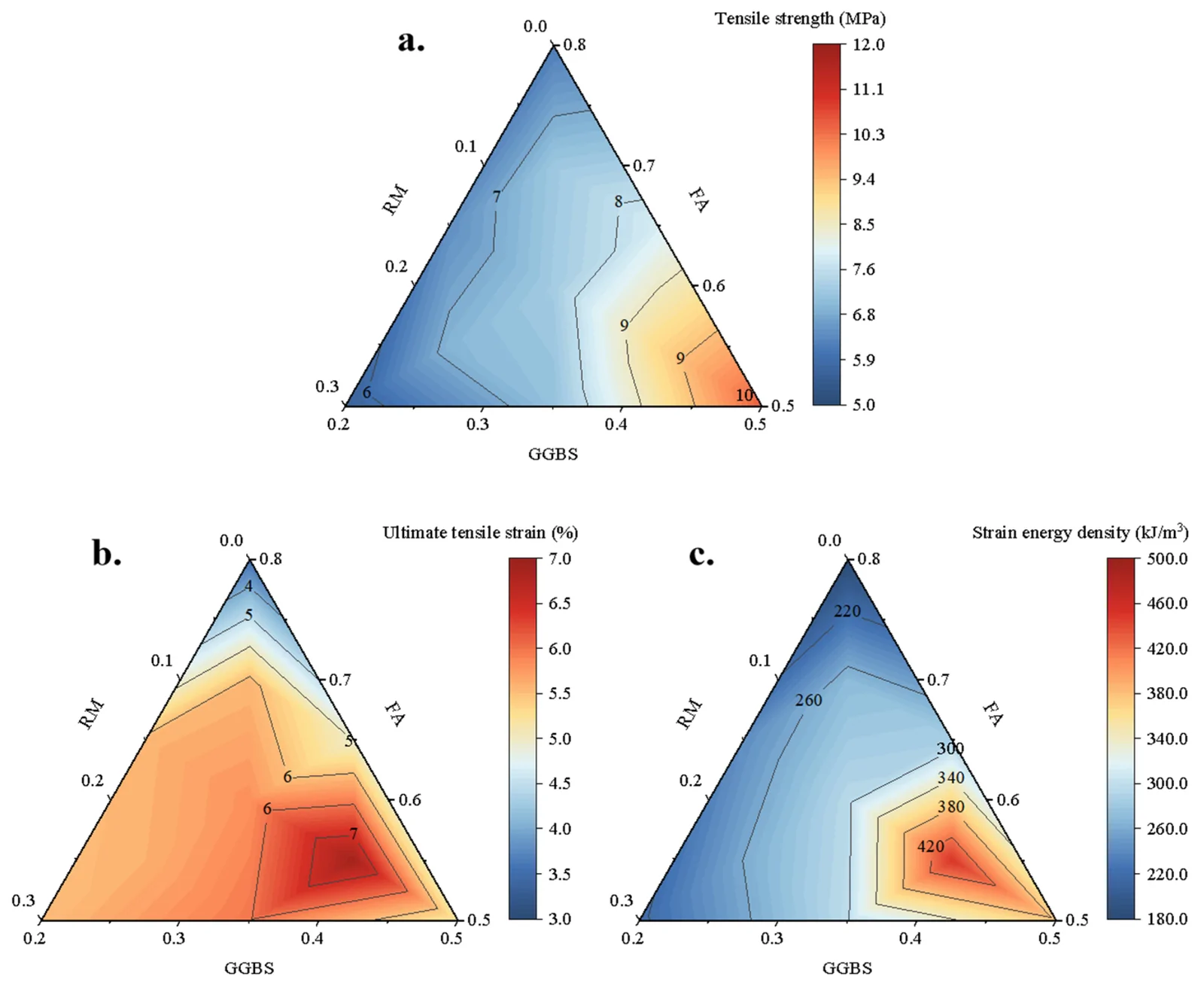
Contour plots of the axial tensile performance indices of the EGC mixtures: (**a**) tensile strength; (**b**) ultimate tensile strain; and (**c**) strain energy density.

Based on the tensile performance parameters listed in Table 5, contour plots were constructed, as shown in Figure 12. By fitting the experimental data, the relationships between the tensile performance indices and the binder composition were obtained, as expressed in Equations (7)–(9):

(7) $$\sigma_{t}=25x_{1}+5x_{2}+49x_{3}-17x_{1}x_{2}-308x_{1}x_{3}-83x_{2}x_{3}+535x_{1}x_{2}x_{3}$$

(8) $$\varepsilon_{t}=-5x_{1}-x_{2}+17x_{3}+35x_{1}x_{2}-136x_{1}x_{3}-22x_{2}x_{3}+332x_{1}x_{2}x_{3}$$

(9) $$g_{se}=447x_{1}-51x_{2}+3258x_{3}+811x_{1}x_{2}-19493x_{1}x_{3}-6265x_{2}x_{3}+39788x_{1}x_{2}x_{3}$$

As evidenced by Table 5 and Figure 12, the tensile strength and ultimate tensile strain of the composites were strongly dependent on the relative proportions of RM, FA, and GGBS in the precursor system. A comparison among G50F50R0, G35F65R0, and G20F80R0 indicates that tensile strength was governed primarily by the GGBS content. As the GGBS proportion increased from 20% to 50%, the tensile strength increased markedly by 71.1%.

At a constant GGBS content of 20%, a moderate increase in the RM proportion, as represented by G20F80R0 and G20F65R15, reduced the resistance of the matrix to first cracking and facilitated microcrack initiation. This promoted the formation of multiple cracks and enhanced the tensile deformation capacity and overall ductility of the composites. As shown in Figure 12, the beneficial effect was most pronounced when the RM content ranged from approximately 3% to 9%. Within this range, RM, FA, and GGBS exhibited a favorable synergistic interaction, enabling more effective stress redistribution under uniaxial tension and delaying crack localization into a single dominant crack. Consequently, the ultimate tensile strain was substantially improved.

These results demonstrate that careful control of the RM content can optimize the ductility of EGC without causing a substantial loss in tensile strength. However, when the RM content became excessive, as in G20F50R30, the tensile performance deteriorated, primarily because of the reduced fiber-bridging capacity.

It is worth noting that, in the composite systems with a constant GGBS content, the influence of RM content on strain energy density exhibited a nonlinear trend. When the RM content increased from 0% to 15%, as represented by G20F80R0 and G20F65R15, the strain energy density increased significantly by 34.2%. However, when the RM content was further increased to 30%, the strain energy density decreased from 242.6 kJ/m^3^ for G20F65R15 to 216.1 kJ/m^3^ for G20F50R30, corresponding to a reduction of 10.9%, although it remained higher than that of G20F80R0 without RM.

The nonlinear variation in strain energy density can be attributed to the competition between matrix weakening and crack-distribution enhancement: moderate heterogeneity promotes multiple microcracking and stress redistribution, whereas excessive weak phases or weak interfaces accelerate crack localization and reduce the effectiveness of fiber bridging [43,44]. Overall, the beneficial mechanical effects of RM incorporation were mainly reflected in the substantial enhancement of tensile deformation capacity and strain energy density. From the perspective of performance optimization, the use of RM in EGC production can therefore be regarded as a feasible approach and an effective pathway for the resource utilization of RM.

### 3.4. Design of Ternary Cementitious System EGC

Based on the above experimental results, mixture optimization of EGC was conducted using the simplex-centroid method. Contour plots were established for four performance indices, namely compressive strength, ultimate tensile strain, tensile strength, and tensile strain energy density. A multi-objective overlay analysis was then adopted according to engineering requirements by superimposing the critical boundary lines of these performance parameters within a unified coordinate system, thereby enabling the coordinated design of the composite mechanical properties.

**Figure 13:**
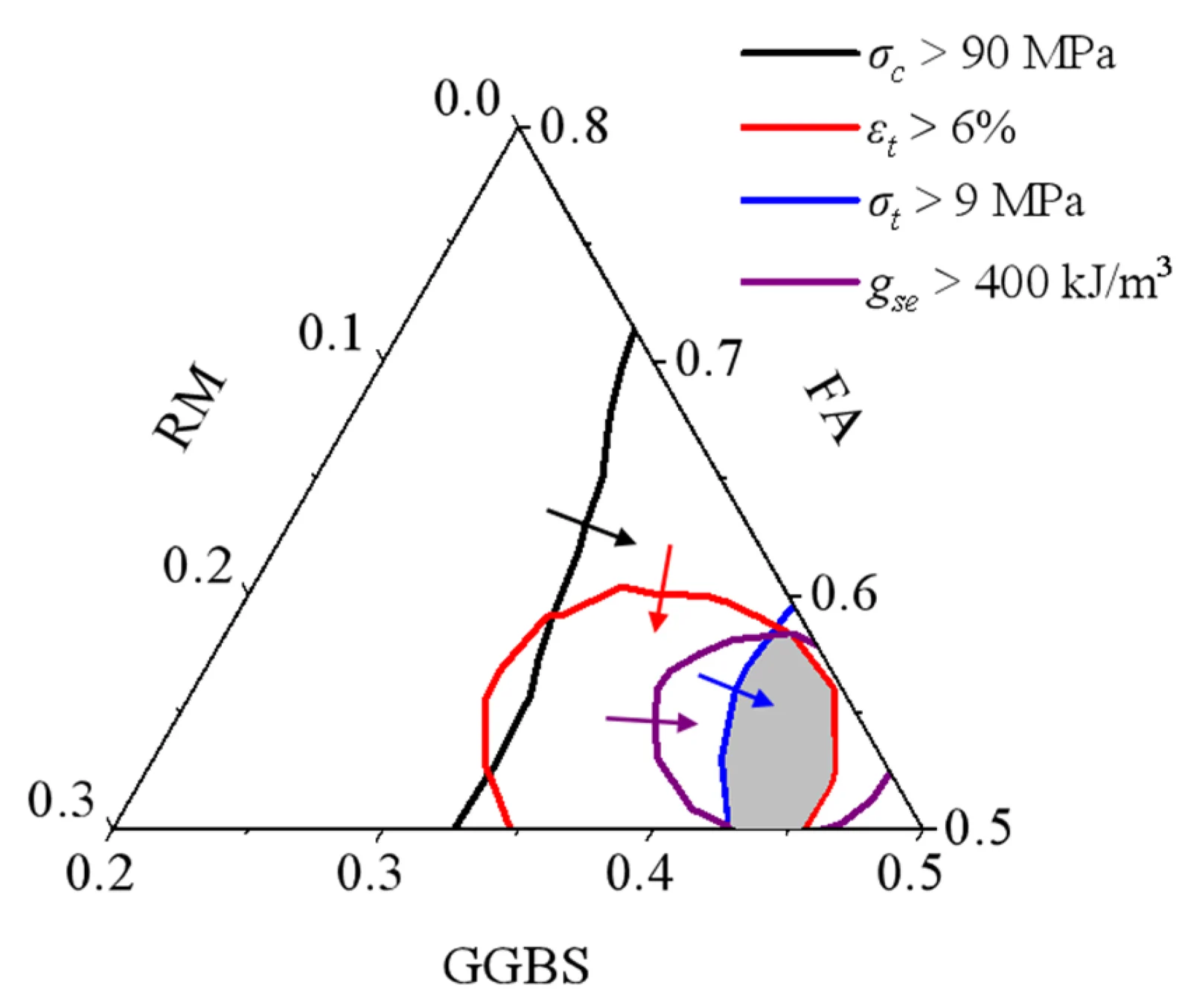
Illustrative case of performance optimization for EGC with a multicomponent binder system.

Figure 13 presents an illustrative optimization case, in which the following criteria were selected as the target thresholds: compressive strength > 90 MPa, ultimate tensile strain > 6.0%, tensile strength > 9 MPa, and tensile strain energy density > 400 kJ/m^3^. The gray shaded region in the figure represents the overlap of the feasible ranges defined by these performance criteria, corresponding to the optimized mixture-design window for this case. As shown, mixture compositions with 40–45% GGBS, 50–60% FA, and 0–8% RM can achieve the coordinated optimization of the target mechanical properties of EGC. The optimized range proposed in Figure 13 is based on the design suggestions derived from the fitting model. It is worth noting that the original design axis point G40F55R5 is precisely located within this optimized range. The actual four performance indicators all meet the target thresholds, providing experimental support for the feasibility of this optimized range.

## 4. Conclusions

This study developed engineered geopolymer composites based on a ternary GGBS–FA–RM binder system. Ten EGC mixtures with different binder proportions were designed using the simplex-centroid method, and their axial compressive and axial tensile properties were experimentally investigated. The feasibility of incorporating RM to optimize the mechanical performance of EGC was evaluated, and the effects of binder composition were systematically clarified. The main conclusions are as follows:

(1) Owing to its rough and porous surface, relatively high specific surface area, and disruption of the original particle-packing structure, RM significantly reduced the flowability of the fresh EGC mixtures as its content increased.
(2) The GGBS content was the dominant factor governing compressive performance. Increasing the GGBS proportion promoted the formation of a denser C–A–S–H gel network, resulting in marked increases in compressive strength and elastic modulus, with a maximum compressive strength of 111.7 MPa. Although RM incorporation reduced compressive strength, a strength of approximately 70 MPa was still maintained at an RM content of 30%. Meanwhile, RM mitigated post-peak brittleness, and the failure mode gradually shifted from longitudinal splitting, characteristic of high-strength specimens, to a more ductile inclined shear failure.
(3) An appropriate RM content effectively reduced the first-cracking strength of the matrix and promoted microcrack initiation. Under fiber bridging, the composites exhibited pronounced strain-hardening and multiple-cracking behavior. RM contents of 5–15% provided the most favorable improvements in crack control and strain energy density. Excessive RM incorporation, such as 30%, diluted the effective cementitious phases with inert constituents, weakened the matrix, widened the cracks, and consequently deteriorated the tensile performance.
(4) By superimposing the performance boundaries of compressive strength, ultimate tensile strain, tensile strength, and strain energy density, the high-strength, high-ductility, and high-energy-absorption characteristics of the composites could be simultaneously optimized.

This study has confirmed the technical feasibility of preparing EGC using RM. However, this research has some limitations that need to be taken into account. The optimized composition range was determined based on the fitting model and multi-objective superimposed analysis. Therefore, subsequent experiments should be conducted on the mixture located at the center of the determined optimal region. Moreover, for the optimized ratio that meets specific engineering requirements, further experimental verification should be carried out before its application in engineering. Relevant work will be carried out in subsequent studies.

## Figures and Tables

**Table 1:** Chemical compositions of GGBS, FA and RM (unit: %).

Oxide	SiO_2_	Al_2_O_3_	CaO	MgO	Fe_2_O_3_	Na_2_O	TiO_2_	K_2_O	Others
GGBS	28.49	15.53	41.97	8.19	0.47	0.51	1.21	0.41	3.22
FA	51.89	31.11	4.88	0.85	4.97	0.46	1.38	2.54	1.92
RM	12.72	21.41	0.63	0.11	48.28	9.76	5.41	0.16	1.52

**Table 2:** Mix proportion (kg/m^3^).

Mix IDs	GGBS	FA	RM	QS	Activator	Water	BaCl_2_	PE Fiber
G50F50R0	606.90	606.90	0	242.76	485.52	93.40	12.14	19.40
G35F65R0	424.83	788.97	0					
G20F80R0	242.76	971.04	0					
G40F55R5	485.52	667.59	60.69					
G25F70R5	303.45	849.66	60.69					
G30F60R10	364.14	728.28	121.38					
G35F50R15	424.83	606.90	182.07					
G20F65R15	242.76	788.97	182.07					
G25F55R20	303.45	667.59	242.76					
G20F50R30	242.76	606.90	364.14					

**Table 3:** Axial compressive properties of the EGC mixtures.

Mix IDs	*f*_c_ (MPa)	*ε*_c_ (%)	*E* (GPa)
G50F50R0	111.7 (1.7)	0.614 (0.033)	20.9 (1.4)
G35F65R0	93.7 (3.4)	0.597 (0.034)	17.8 (0.5)
G20F80R0	77.0 (2.4)	0.707 (0.033)	13.4 (0.6)
G40F55R5	95.3 (6.5)	0.552 (0.022)	19.6 (2.3)
G25F70R5	85.4 (1.0)	0.752 (0.019)	15.1 (1.2)
G30F60R10	89.0 (2.9)	0.658 (0.016)	16.2 (1.4)
G35F50R15	93.2 (4.7)	0.581 (0.030)	17.4 (0.2)
G20F65R15	66.6 (6.1)	0.678 (0.061)	12.0 (0.8)
G25F55R20	77.3 (2.9)	0.618 (0.010)	14.7 (0.2)
G20F50R30	70.3 (2.4)	0.656 (0.043)	13.4 (0.7)

Note: Values in parentheses represent the standard deviations of the experimental results.

**Table 4:** Crack parameters of EGC.

Mix IDs	*w_c_* (μm)	*N_c_*	*x_c_* (mm)
G50F50R0	48.1 (4.5)	43.7 (2.5)	1.84 (0.11)
G35F65R0	50.1 (12.3)	41.7 (4.7)	1.94 (0.21)
G20F80R0	51.8 (4.0)	35.0 (3.6)	2.30 (0.23)
G40F55R5	50.7 (4.8)	53.3 (3.1)	1.50 (0.09)
G25F70R5	52.1 (4.2)	49.3 (6.8)	1.64 (1.21)
G30F60R10	53.7 (3.3)	48.7 (2.1)	1.65 (0.07)
G35F50R15	48.6 (10.6)	56.3 (8.3)	1.44 (0.23)
G20F65R15	47.9 (10.5)	44.7 (3.5)	1.80 (0.14)
G25F55R20	42.7 (7.4)	56.3 (6.7)	1.43 (0.16)
G20F50R30	56.1 (7.0)	51.3 (1.5)	1.56 (0.05)

**Table 5:** Tensile parameters of EGC.

Mix IDs	*σ_tc_* (MPa)	*σ_tu_* (MPa)	*ε_tu_* (%)	*g_se_* (kJ/m^3^)
G50F50R0	3.24 (0.16)	10.47 (0.32)	5.16 (0.08)	376.4 (15.0)
G35F65R0	3.25 (0.42)	7.88 (0.22)	4.97 (0.11)	286.1 (9.8)
G20F80R0	2.52 (0.55)	6.12 (0.41)	3.54 (0.81)	180.8 (44.7)
G40F55R5	2.91 (0.24)	9.06 (0.54)	6.90 (0.51)	451.6 (47.4)
G25F70R5	1.75 (0.20)	7.19 (0.32)	5.56 (0.28)	270.1 (12.2)
G30F60R10	1.64 (0.09)	7.26 (0.35)	5.81 (0.31)	294.7 (17.1)
G35F50R15	1.61 (0.28)	7.06 (0.37)	5.98 (0.20)	298.0 (23.2)
G20F65R15	2.29 (0.10)	6.38 (0.39)	5.58 (0.17)	242.6 (13.7)
G25F55R20	2.50 (0.35)	6.90 (0.14)	5.61 (0.27)	260.4 (16.6)
G20F50R30	2.03 (0.16)	5.60 (0.29)	5.51 (0.03)	216.1 (15.3)

Note: Values in parentheses represent the standard deviations of the experimental results.

## Data Availability

The data presented in this study are available upon request from the corresponding author. The data are not publicly available due to confidentiality issues.
